# Tamoxifen resistance in early breast cancer: statistical modelling of tissue markers to improve risk prediction

**DOI:** 10.1038/sj.bjc.6605627

**Published:** 2010-05-11

**Authors:** M R Baneshi, P Warner, N Anderson, J Edwards, T G Cooke, J M S Bartlett

**Affiliations:** 1 Centre for Population Health Sciences, University of Edinburgh, Teviot Place, Edinburgh, UK; 2 Department of Biostatistics and Epidemiology, Health School, Kerman Medical University, Kerman, Iran; 3 Section of Surgery, Division of Molecular Pathology and Cancer Sciences, Level 3, McGregor Building, Western Infirmary, Glasgow G11 6NT, UK; 4 Endocrine Cancer Group, Edinburgh Cancer Research Centre, Western General Hospital, University of Edinburgh, Crewe Road South, Edinburgh EH4 2XR, UK

**Keywords:** breast cancer, tamoxifen resistance, statistical model, HER2 signalling

## Abstract

**Background:**

For over two decades, the Nottingham Prognostic Index (NPI) has been used in the United Kingdom to calculate risk scores and inform management about breast cancer patients. It is derived using just three clinical variables – nodal involvement, tumour size and grade. New scientific methods now make cost-effective measurement of many biological characteristics of tumour tissue from breast cancer biopsy samples possible. However, the number of potential explanatory variables to be considered presents a statistical challenge. The aim of this study was to investigate whether in ER+ tamoxifen-treated breast cancer patients, biological variables can add value to NPI predictors, to provide improved prognostic stratification in terms of overall recurrence-free survival (RFS) and also in terms of remaining recurrence free while on tamoxifen treatment (RFoT). A particular goal was to enable the discrimination of patients with a very low risk of recurrence.

**Methods:**

Tissue samples of 401 cases were analysed by microarray technology, providing biomarker data for 72 variables in total, from AKT, BAD, HER, MTOR, PgR, MAPK and RAS families. Only biomarkers screened as potentially informative (i.e., exhibiting univariate association with recurrence) were offered to the multivariate model. The multiple imputation method was used to deal with missing values, and bootstrap sampling was used to assess internal validity and refine the model.

**Results:**

Neither the RFS nor RFoT models derived included Grade, but both had better predictive and discrimination ability than NPI. A slight difference was observed between models in terms of biomarkers included, and, in particular, the RFoT model alone included HER2. The estimated 7-year RFS rates in the lowest-risk groups by RFS and RFoT models were 95 and 97%, respectively, whereas the corresponding rate for the lowest-risk group of NPI was 89%.

**Conclusion:**

The findings demonstrate considerable potential for improved prognostic modelling by incorporation of biological variables into risk prediction. In particular, the ability to identify a low-risk group with minimal risk of recurrence is likely to have clinical appeal. With larger data sets and longer follow-up, this modelling approach has the potential to enhance an understanding of the interplay of biological characteristics, treatment and cancer recurrence.

Endocrine-targeted therapy remains one of the most successful systemic treatment options for the approximately 80% of patients diagnosed with ER-positive early breast cancer (Miller *et al*, 2007; Bartlett *et al*, 2007). However, it has been shown that many patients relapse and die from breast cancer, despite the relative efficacy of current endocrine treatment modalities (Abe *et al*, 2005). Recent advances, including the third generation aromatase inhibitors, have not dramatically altered this figure (Hughes-Davies *et al*, 2009). Conversely, a significant proportion of women with ER positive cancer are at a low risk of breast cancer relapse, even when not treated with adjuvant endocrine therapy (Abe *et al*, 2005).

Currently, treatment selection for breast cancer is guided predominantly by patient prognosis, using classical pathological assessment of tumours to measure risk (Pinder *et al*, 1995; Elston *et al*, 1999). Endocrine therapy is offered to the majority of patients with ER*α-*positive breast cancers, with higher risk patients being more likely to receive aromatase inhibitors and possibly chemotherapy. Two fundamental changes in the understanding of the underlying biology of breast cancers challenge this approach. First, the molecular differences that exist between breast cancers (Perou *et al*, 2000; Pollack *et al*, 2002; Desmedt *et al*, 2004) support treating different molecular subtypes on the basis of their biology and pathology rather than on pathology alone. Second, clear evidence that molecular subtypes of cancer respond differently to different therapeutic options challenges the ‘one size fits all’ approach to chemotherapy in cancer (Hayes *et al*, 2007; Bartlett *et al*, 2008, 2009a, 2009b, 2010; Pritchard *et al*, 2008; Ellis *et al*, 2009).

Several different approaches have been applied to the subclassification of breast cancers on the basis of genomic (Pollack *et al*, 2002), transcriptomic (Perou *et al*, 2000) and immunohistochemical (El Rehim *et al*, 2004) techniques. No clear evidence has yet emerged as to the superiority of one approach over another. We are pursuing a functional approach for the stratification of breast cancers, seeking to ‘translate’ current knowledge of drug resistance pathways to the identification of subgroups with low, moderate and high risk of relapse after treatment with particular therapeutic agents.

The Nottingham Prognostic Index (NPI) combines information on nodal status, tumour grade and tumour size in untreated breast cancer patients by means of a Cox regression model, to produce an estimate of the risk of cancer recurrence that can be used to classify patients into risk groups (Haybittle *et al*, 1982) for treatment selection. This model has been widely validated and is now central to the risk stratification of patients with breast cancer across the United Kingdom. However, on the basis of analyses of nearly 10 000 patients, it has been concluded that NPI is not capable of identifying a low enough risk group to warrant a recommendation of no treatment (Balslev *et al*, 1994). Therefore, there is a need for additional prognostic factors to improve the precision of prediction.

Similar models, notably ‘Adjuvant! Online’, were derived in an analogous manner and are used across Europe and the United States. These models, particularly Adjuvant! Online, have sought to adapt to modern practice by integrating ER*α* with clinical trial data to select appropriate therapies for patients. However, such approaches tend to select only the single most powerful predictive biomarker for inclusion within the model.

We have, over the past few years, carefully explored the role of a large number of candidate predictive biomarkers in a selected cohort of tamoxifen-treated ER*α-*positive breast cancer patients (Kirkegaard *et al*, 2005, 2007; Tovey *et al*, 2005, 2006b; Naresh *et al*, 2006; Cannings *et al*, 2007; McGlynn *et al*, 2009). In so doing, we have mapped the expression of markers that appear independently predictive of response to tamoxifen (Kirkegaard *et al*, 2005, 2007; Tovey *et al*, 2005), including AIB1, HER2 and AKT. In this way, we have identified a novel predictive biomarker panel with the potential to improve selection of patients with ER*α-*positive breast cancers who are likely to respond well to tamoxifen and, potentially, to other endocrine therapies.

In this study, we explore the potential of combining biomarker data and clinical variables to develop an enhanced prognostic index for breast cancer recurrence.

## Materials and methods

### Patients

Study subjects comprised 401 ER-positive patients diagnosed between 1983 and 1999 at the Glasgow Royal Infirmary (McGlynn *et al*, 2009). The median follow-up time was 6.16 years and all patients received tamoxifen with a median treatment duration of 5 years. All patients were treated by surgery with curative intent and received tamoxifen after surgery; 73 (18%) were aged under 50 years at diagnosis. With regard to other adjuvant treatments, 74 (28%) patients received radiotherapy only, 61 (25%) received chemotherapy only and 40 (10%) received both (chemotherapy information was unknown for three patients, one of whom received radiotherapy and is counted among the 74).

By the end of follow-up, there had been 74 deaths among the 112 recurrences, and 84 of the recurrences occurred while the patient was still receiving tamoxifen treatment.

### Variables

Data from previous analyses, quality assured by dual scoring (Kirkegaard *et al*, 2005, 2006, 2007, 2008; Tovey *et al*, 2005, 2006a, 2006b; Cannings *et al*, 2007), were considered for inclusion in the model (72 variables relating to 41 biomarkers). Nuclear, cytoplasmic and membrane expressions were scored according to the observed cellular distribution of markers and were analysed separately. Membrane expression was analysed for p118ER*α*, p167ER*α*, EGFr, HER2, phosphoHER2, HER3 (m), HER4-ICD (intracellular domain) and HER4 ECD (extracellular domain). Cytoplasmic expression was analysed for ER*α*, ER*β*, p118ER*α*, p167ER*α*, phosphoHER2, HER3, HER4-ICD, HER4 ECD, hRAS, nRAS, kRAS, RAF1, p259-RAF1, p338-RAF1, rKip, TES, AKT1, AKT2, AKT3, panAKT, p473AKT, p308AKT, mTOR, phospho-mTOR, p389-p70S6k, Tace, Tacep, MAPK, phosphoMAPK, PTEN, Bcl2, Bax, Bad, p112-Bad and Bcl-xl. Nuclear expression was analysed for ER*α*, ER*β*, PgR, p118ER*α*, p167ER*α*, phosphoHER2, HER3, HER4-ICD, HER4 ECD, hRAS, nRAS, kRAS, RAF1, p259-RAF1, p338-RAF1, rKip, TES, AKT1, panAKT, p473AKT, p308AKT, MAPK, phosphoMAPK, PTEN and AIB1. In addition, gene amplification and copy number for HER2 and AIB1 and TUNEL analysis of apoptosis were analysed. Four clinical variables were also offered to the model, namely, nodal status, grade (Bloom and Richardson), tumour size and age.

### Outcomes studied

The primary outcome was recurrence-free survival (RFS), with secondary outcomes being overall survival (OS) and remaining recurrence free while on tamoxifen treatment (RFoT), as previously defined (Kirkegaard *et al*, 2005; Tovey *et al*, 2005, 2006a, 2006b). Separate models were developed for RFS and RFoT. Kaplan–Meier (K–M) curves are presented for RFS and RFoT in relation to risk groups obtained from the models developed for these end points, and also for OS in relation to the patient risk groupings obtained from the RFS and RFoT models.

### Statistical modelling

**Figure 1:**
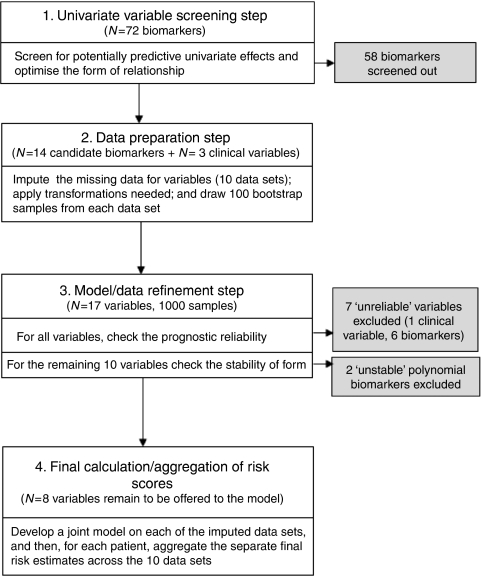
Process of development of RFS model.

Regression risk modelling techniques perform best when there are relatively large numbers of events and comprehensive data for all biomarkers (Peduzzi *et al*, 1995). However, the number of events in this cohort is not large (particularly for recurrence on tamoxifen). By their nature, biomarkers are prone to missing values, and it tends to be that the distribution of biomarker expression is positively skewed (and hence relationship with recurrence likely to be nonlinear) (Royston and Sauerbrei, 2008). To address these challenges, we developed a four step modelling approach (Figure 1).

#### Step 1: Screening and choice of risk function

Our screening process had two inter-linked aims: (i) to select variables to be offered as candidate variables to the multivariate model, those demonstrating univariate association with outcome (recurrence or recurrence while on tamoxifen); and (ii) to identify the best form of association with recurrence (linear, polynomial, threshold or non-ordinal). To avoid screening of variables that are potentially important in the final multivariate model, the screening *P*-value threshold was set to the equivalent of *P*=0.1 in a standard univariate Cox model (i.e., *P*-value <0.1 in fractional polynomial (FP) (explained in the next paragraph); <0.005 in the minimum *P*-value method; or <0.025 in ‘non-ordinal quartile dichotomisation’ methods.)

For each biomarker in turn, we first applied second-degree FP (FP2) regression to detect polynomial or linear associations. FP1 functions are power transformations modelling *X*^*p*^ rather than the variable *X* (where *P*= −2, −1, 0, 0.5, 1, 2, 3), whereas the FP2 form is an extension to β_1_ X^*p*_1_^ + β_2_ X^*p*_2_^ (Royston and Altman, 1994). The simpler FP1 or linear form was selected if it provided an adequate fit. For any variable not included by the FP method, the existence of a threshold effect (in which expression at or above a specified level predicts outcome) was checked by a minimum *P*-value method (Clark *et al*, 1993). Any variable remaining unselected was then checked for non-ordinal effects by comparing cases with expression of the biomarker ranging between two neighbouring quartiles *vs* the remaining cases; or middle two quartile ranges *vs* the remaining; or first and third quartile ranges *vs* the remaining (four comparisons for each biomarker).

#### Step 2: Data imputation and selection of bootstrap samples

For all candidate variables, we imputed missing data using MICE (multivariate imputations by chained equations), a probability-based simulation technique that takes into account imputation uncertainty (Schafer, 1999). This is an iterative process in which missing data for a variable are estimated using its imputation model and, in turn, these data are used in the estimation of missing data for other variables. In accordance with usual practice, we imputed 10 values for each missing value, thus creating 10 imputed data sets. Any transformations needed to achieve optimum form, as identified during screening in step 1, were then applied to relevant variables in each of the 10 data sets. For subsequent checking of stability/reliability, 100 bootstrap samples were drawn from each imputed data set, resulting in 1000 ‘sample’ data sets.

#### Step 3: Refinement of model to eliminate unreliable and unstable predictors

A stable effect/form was defined as one occurring in at least 50% of the 1000 sample data sets. First, we checked stability of threshold/non-ordinal effects. Stable threshold/non-ordinal variables, variables screened in step 1 as having linear/polynomial association, and clinical variables were then subjected to predictive model fitting using backward elimination, with the threshold for removing variables *P*=0.05. This was undertaken separately for each of the 1000 sample data sets. An MFP (multivariate fractional polynomial) multivariate modelling approach was used, which, after fitting of linear factors, ascertains whether the model fit could be improved by using a polynomial form for any of the linear variables (Royston and Sauerbrei, 2008). For classification as unreliable variables, the inclusion frequency of each variable across all 1000 models was checked, and if less than 50%, the variable was deemed ‘unreliable’, as per Sauerbrei's algorithm (Sauerbrei and Schumacher, 1992), and excluded. Furthermore, for continuous biomarkers, if the form of risk function was unstable across bootstrap sample models (as defined above), the variable was dropped.

#### Step 4: Aggregation of results

Using only the ‘stable’ and ‘reliable’ variables identified in the previous step, a final model was then fitted to each of the 10 imputed data sets. Applying Rubin's rule, coefficients for these 10 models were then averaged across models, and standard errors were combined (Rubin, 1976), and an aggregate risk score was obtained for each patient by averaging his/her risk scores across the models obtained for each of the 10 imputed data sets.

For the RFS model, four equally sized risk groups were created by setting the cutoff points at three-quartiles of the distribution of the corresponding aggregate patient risk scores. Given that the RFoT model was based on fewer events, three risk groups were created by applying two tertile cutoff points to the distribution of corresponding aggregate patient risk scores.

### Model performance

The final aggregated model(s) for RFS and RFoT were compared with NPI in terms of its discrimination (C-index) and ability to predict disease relapse (Nagelkerke *R*^2^) (Harrell *et al*, 1996). The C-index is a generalisation of the area under the ROC curve and quantifies the ability to distinguish low- and high-risk patients. This statistic varies between 0.5 (no better than chance) and 1, with values near 1 indicating high discrimination power. The Nagelkerke *R*^2^ varies between 0 and 1, which indicates, respectively, very poor and very high predictive ability (Harrell *et al*, 1996). Kaplan–Meier survival curves have been plotted to allow a visual comparison of event-free survival curves within risk groups for the models being compared.

We also assessed the extent to which each biomarker model classified patients into more appropriate risk groups compared with NPI (Pencina *et al*, 2008). For recurrence-free patients and in those in whom disease recurred, the method separately considers the joint distribution of patients into risk groups by the standard and new models being compared, quantifying ‘improvement’ in risk group classifications; for recurrence cases, the new model classifies them as higher risk, and for recurrence-free cases, as lower risk, compared with the standard model. As our RFS model classified patients into four equally sized risk groups, a fair application of this method required a comparable division of NPI risk scores (splits at quartiles of NPI risk scores were therefore used, i.e., 3.3, 4.2 and 4.8). A similar approach was used for the RFoT model, using tertile-split patients.

For both models, the actuarial event-free rate in the lowest risk group is reported for up to 3, 5, 7 and 10 years of follow-up. For comparisons of model ability to identify low-risk patients, event-free rates at 7 years are used, because follow-up data to 10 years are as yet sparse.

### Software

Analyses were performed using SPSS (V.13) (SPSS, Chicago, IL, USA) and R software using MFP (Ambler and Benner, 2008), Maxstat (Horton, 2007), MICE (Van Buuren and Oudshoorn, 2007), Mitools (Lumley, 2008), Hmisc (Harrell, 2008b) and Design libraries (Harrell, 2008a).

## Results

### RFS modelling

Table 1 shows the 14 biological variables selected by univariate screening (step 1); for nine variables, the relationship with recurrence was linear, for two the best functional form was expressed by an FP2 model and a reciprocal square transformation (FP1) was used for nuclear PTEN. For nuclear phospho-MAPK (pMAPK), a threshold effect was identified (using a histoscore of 104) and for nuclear AKT1 (AKT1), a non-ordinal association was detected (the group with value between the second and third quartile, differing in recurrence from the rest). The frequency of missing values for candidate variables ranged between 1.2 and 11%, with the average missing rate being 5.2%. In all, 262 patients (65% of cases) had complete data on all selected biomarkers and clinical variables.

**Figure 2:**
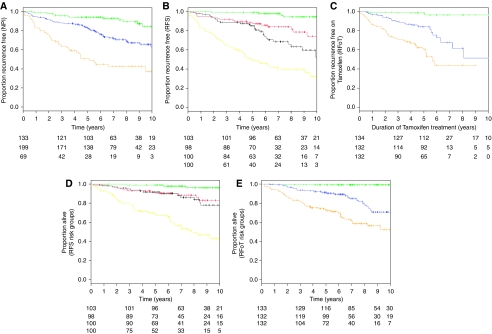
Kaplan–Meier curves for the following: RFS by NPI grouping (**A** top left); RFS by RFS biomarker model grouping (**B** top middle); RFoT by RFoT biomarker model grouping (**C** top right); OS by RFS biomarker model grouping (**D** bottom left); and OS by RFoT biomarker model grouping (**E** bottom right).

The inclusion frequency for all 17 variables offered to multivariate RFS models is given in the first column in Table 2. The final multivariate model for RFS retained six biomarkers and two clinical variables as shown in the first panel of Table 2, together with aggregated hazard ratios (as described in Materials and Methods section). It can be seen that the stability of threshold effect for pMAPK and the non-ordinal effect for AKT1 were confirmed across bootstrap samples. Kaplan–Meier curves for RFS are presented in Figure 2A, using standard NPI risk groups, and in Figure 2B using the four risk groups derived from our RFS biomarker model. The main difference between the two plots is that the lowest risk group using the RFS model displays less recurrence than the lowest risk NPI group.

### Modelling RFoT

Three patients with missing values on duration of tamoxifen treatment could not be included in the analysis. Table 2 (right-hand panel) presents the results for RFoT modelling. Univariate screening with respect to RFoT selected 17 candidate variables, the inclusion frequencies of which are reported. Only five of these were included in the final model, and their estimated hazard ratios are reported. Figure 2C shows K–M curves for risk groups derived from our RFoT model, and it can be seen that there is very little recurrence in the lowest risk group.

### Overall survival by risk groupings of RFS and RFoT models

Kaplan–Meier curves for OS, using the RFS and RFoT model risk groupings, are presented in Figures 2D and E, respectively. These suggest that the RFS and RFoT models can discriminate patients with a low and high risk of death.

### Performance of models

The RFS biomarker model had a higher discrimination ability than NPI (C-index 79 *vs* 72% for NPI). The corresponding figures for the RFoT model were 78 and 75%, respectively. There was a similar finding for predictive ability (*R*^2^: 27 *vs* 14% for RFS and 19 *vs* 17% for RFoT).

Estimated 7-year RFS in the lowest risk group was 95% for the RFS biomarker model (four-group stratification), whereas it was 89% for NPI (standard three groups). The corresponding rates at 10 years were 95 and 79%, respectively.

Recurrence free while on tamoxifen treatment rates were compared at 5 years, because only a small proportion of patients received tamoxifen for more than 5 years. Estimated 5-year RFoT in the lowest risk group was 97% for the biomarker model (also three groups) and 94% for NPI (standard grouping).

**Figure 3:**
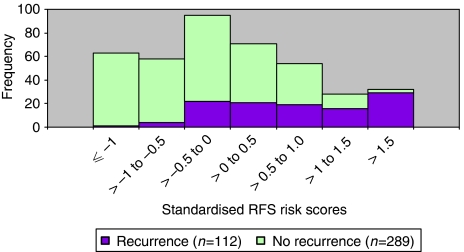
Distribution of the estimated standardised risk scores for recurrence, with indication within each bar of the number of recurrences observed.

Furthermore, as shown in Figure 3, out of 63 patients with standardised risk scores of one or less, only a single patient recurred giving 7-year RFS of 98%, whereas out of 32 patients with risk scores exceeding 1.5, 29 patients recurred, giving a 7-year RFS of only 14%.

If we compare risk group classifications overall, for our RFS model compared with NPI, then of 112 recurrent cases, 34 (30%) were more appropriately classified by RFS (i.e., to a higher risk group), whereas 18 (16%) were assigned to a less appropriate group (lower), giving a net gain in classification appropriateness of 14% by RFS (*P*<0.01). For the 289 recurrence-free cases, the corresponding changes in classification were 90 (31%) and 78 (27%), giving a net gain in classification appropriateness of 4% (*P*=0.17). For the RFoT model, the net gains in classification appropriateness in recurred and non-recurred subgroups were 7.1 and 1.3%, respectively (*P*>0.05 for both).

## Discussion

Endocrine therapy, using either tamoxifen or aromatase inhibitors, remains the most successful systemic treatment of early breast cancer. Significant improvements in recurrence-free and OS are achieved by treating women with hormone receptor-positive disease with ER-targeted therapies for 5–10 years (Abe *et al*, 2005). However, many women do not require endocrine therapy, achieving sufficient disease control from surgery and local radiotherapy (Abe *et al*, 2005). A further group may derive minimal additional benefit over that achieved with tamoxifen treatment if treated with aromatase inhibitors and/or chemotherapy (Abe *et al*, 2005; Miller *et al*, 2007; Hughes-Davies *et al*, 2009). The challenge is to devise prospective diagnostic approaches to stratify women for appropriate adjuvant management, including identification of those women who require no adjuvant hormonal or chemotherapy.

Using retrospective statistical modelling of molecular analysis of intracellular signalling pathways, we developed an algorithm that allows the calculation of risk of recurrence for early breast cancers treated with tamoxifen. Individual risk scores were calculated using a simple panel of six immunohistochemical markers (in addition to tumour size and nodal status), and when patients were stratified by risk into four quartiles, marked differences in group relapse rates were observed.

Among patients in the lowest risk group by the RFS model, the estimated 7-year RFS rate was 95%, whereas in the highest risk group, it was only 40% (Figure 2). When we moved the cutoffs to create risk groups that mirrored the numbers of patients in NPI risk groups for our cohort (133 lowest-risk, 199 intermediate-risk and 69 highest-risk group), the estimated 7-year RFS remained well separated, at 95% (95% CI: 91, 99%) and 34% (95% CI: 22, 46%) in the lowest and highest risk groups, respectively.

Low-risk patients could potentially avoid systemic treatment, perhaps those with risk scores no greater than one, with a 98% RFS rate at 7 years. Conversely, higher risk patients might well be candidates for additional treatment, including chemotherapy or other adjuvant treatment options. The application of individual risk scores such as those that have been derived in our models might become a strong driver for the implementation of biological risk prediction.

The advantage of our biomarker model in the stratification of a group with a low risk of recurrence seems to increase with duration of follow up: the RFS rate at 5-year follow-up was 98% (*vs* 94% by NPI grouping) and at 7 years was 95% (*vs* 89%). This is similar to the prediction achieved by complex multigene PCR-based assay systems (Paik *et al*, 2004). However, an important difference is that immunohistochemical assays are more readily applied to routine pathological assessments than complex multigene panels, and are also potentially significantly more cost-effective (Paik *et al*, 2004).

The model developed provides a significant improvement over conventional prognostic models such as NPI. The limitations of NPI have been recognised for some time and novel modelling approaches, including Adjuvant ! Online, have sought to incorporate biological (ER*α*) and clinical risk markers. However, such biological modelling is incomplete and further attempts at refining the integration of biological and clinical risk markers are required. One of the limitations of the current approach is the use of a tamoxifen-treated cohort; hence, a further analysis of untreated patient cohorts is required to fully validate the model. Use of historical cohorts is often criticised because improvements in screening, surgery and radiotherapy have improved prognosis in recent years, and this might affect prognosis differentially by patient factors. However, there is a conflict between the use of cohorts of patients who have received first-line treatment reflecting contemporary practice in surgical and radiotherapy techniques, but which are relatively recent cases and will thus have a short follow-up, and historical data sets with a longer follow-up. Neither approach truly investigates the natural history of the disease, and both approaches accept the interpretative compromises that are integral to the study population used.

Although the NPI model is very useful, it was developed using a limited range of risk factors. New biological developments allow us to measure functional features of tumours, so that we have a rich array of biomarkers with potential relevance to cancer progression. To circumvent the risk of an overfitted model, which can arise when there are excess potential explanatory variables, as is often the case with biomarkers, we have used bootstrap sampling to refine the models by excluding variables with an unstable form or those that are unreliably included as necessary for prediction. Recent methodological developments also allow modelling gains through detection of an optimum form of association, and provide powerful multiple imputation methods to salvage as much predictive information as possible from cases with missing data. Our biomarker-based predictive tool has the potential for future application in the selection of patients for conservative *vs* aggressive adjuvant treatment. In addition, the results and models reported here can contribute to the generation of hypotheses with regard to the mechanisms that might underlie the differences in recurrences observed.

In the case of the RFoT model, there was a smaller number of events in the analysis (84 *vs* 112); hence, the power was lower compared with the RFS model. This, together with the very limited number of patients with tamoxifen treatment exceeding 8 years, and the potential bias inherent in decisions to proceed or not with tamoxifen therapy (biased censoring), signifies that, at this stage, caution is required if interpreting the model beyond 5 years. Although we applied stringent checks on internal validity of models, the lower power of the model might explain some differences in biomarker selection, compared with other published research. Of variables previously identified as predicting recurrence while on tamoxifen (AIB1, HER2 and AKT) (Kirkegaard *et al*, 2005, 2007; Tovey *et al*, 2005), only HER2 was included in our RFoT multivariate model. This may reflect the fact that HER2 remains the dominant driver in endocrine resistance, and may also reflect the relatively small number of events (84).

One of the interesting differences between the RFS and RFoT models developed on the basis of our data is the inclusion of HER2 as a risk factor only in the latter model. This may reflect the relationship between HER2 overexpression and increased risk of early relapse, or a specific interaction with tamoxifen therapy (resistance). Alternatively, the greater power of the RFS analysis (112 events relative to 84 for RFoT) might have enabled the identification/retention of some other variable(s), which together could provide a better predictive information than HER2, and retained these in the RFS model in place of HER2. Perhaps the RFoT model, without this additional power, had to manage with one variable, which was HER2. Further work using larger sample sizes will be required before the explanation can be clarified.

Several different approaches have been taken for the development of ‘risk’ signatures in early breast cancer. The Mammostrat (Ring *et al*, 2006) panel is based on a functional expression array analysis of different pathways involved in breast cancer recurrence in the absence of adjuvant treatment, which uses five immunohistochemical markers to generate a risk score. The Oncotype Dx test (Paik *et al*, 2004) and MammaPrint are multigene signature-seeking tests, derived in a similar manner and aiming to predict outcome during tamoxifen therapy. Further studies on this marker panel suggest that it may be broadly prognostic, rather than predictive. Our approach has been to use functional markers of key molecular pathways of tamoxifen resistance to seek to identify a panel that can select patients who may either derive sufficient benefit from treatment with tamoxifen alone, or for whom withdrawal of adjuvant therapy, which is moderately toxic, may pose minimal risk. As with all approaches, this has limitations and therefore future analyses should explore additional markers, including perhaps markers of proliferation in addition to those suggested above. A further validation of our current approach is also required and during such a process, direct comparison with similar panels, such as Mammostrat, Oncotype Dx and MammaPrint, would be of value.

NPI is the recognised tool for risk prediction in the United Kingdom. In this study, we showed that TMA variables can add value to clinical predictors. Our model demonstrated significantly better risk group classification performance than NPI, particularly for patients who will go on to experience recurrence. In particular, the lowest risk group identified, comprising a quarter of all patients, showed a high recurrence-free rate (95% at 7 years). This has clinical potential, in that it suggests that such patients might be spared additional treatments without undue risk of recurrence.

## Figures and Tables

**Table 1:** Univariate screening step for RFS model: variables and form of risk function selected

**Variable**	**Form of risk function**	**Number (%) of cases with available data**	**Univariate association with recurrence *P*-value**
*Tissue marker variables*			
Nuclear staining for AKT1 histoscore	Non-ordinal	396 (99)	0.003
Cytoplasmic staining for AKT2 histoscore	Linear	387 (97)	0.06
mTOR histoscore	Linear	379 (95)	0.06
Phospho mTOR histoscore	Linear	390 (97)	0.02
PTEN nuclear histoscore	Polynomial (FP1)	373 (93)	0.02
Phospho-MAPK nuclear IHC histoscore	Threshold (Optimal split=104)	381 (92)	0.003
Phospho Raf (ser338) nuclear histoscore	Linear	357 (89)	0.002
Phospho Raf (ser338) cytoplasmic histoscore	Linear	357 (89)	0.01
Mapk p42/44 cytoplasmic histoscore	Linear	376 (94)	0.01
Cytoplasmic KRAS histoscore	Polynomial (FP2)	387 (97)	<0.001
PgR nuclear histoscore	Linear	387 (97)	0.007
Tunel data	Linear	362 (90)	0.07
Phospho HER2 nuclear histoscore	Linear	376 (94)	0.07
Nuclear RKIP histoscore	Polynomial (FP2)	387 (97)	<0.001
			
*Clinical variables*			
Pathological tumour size	Linear	379 (95)	<0.001
Bloom and Richardson Grade	Linear	390 (97)	<0.001
Nodal status	Linear	368 (92)	<0.001

**Table 2:** Multifactorial RFS and RFoT models; relative frequency of covariate inclusion (in 1000 bootstrap samples)

	**RFS model (112 events) Median follow-up=6.2 (IQR 4.4–8.8) years**			**RFoT model (84 events) Median follow-up=5.0 (IQR 4.0–6.0) years**		
**Variable**	**HR^a^ (95% CI)**	***P*-value**	**Inclusion frequency (%)**	**HR (95% CI)**	***P*-value**	**Inclusion frequency (%)**
Nodal status	1.82 (1.38, 2.40)	<0.001	98.0	2.17 (1.57, 2.99)	<0.001	100
Tumour Size (cm)	1.21 (1.10, 1.31)	0.001	95.2	1.20 (1.10, 1.30)	0.001	87.0
Cytoplasmic kRAS^b^	6.05 (2.23, 16.44)	<0.001	81.6	b		66.0
Tunel	1.49 (1.23, 1.81)	<0.001	85.1	c		
Nuclear Akt1	0.54 (0.36, 0.82)	<0.001	92.3	c		
Phospho mTOR	0.33 (0.19, 0.59)	<0.001	79.1	0.55 (0.33, 0.94)	0.03	72.0
Phospho Raf (ser338) cytoplasmic	2.12 (1.07, 4.02)	0.03	70.8	a		14.2
Phospho MAPK nuclear	2.80 (1.72, 4.57)	<0.001	79.0	c		
PgR nuclear	a		44.0	a		15.4
PTEN Nuclear	b		59.5	b		85.0
Nuclear rKIP	b		55.5	a		13.5
Phospho Raf (ser338) nuclear	a		15.6	2.43 (1.16, 5.13)	0.02	59.4
Grade	a		22.0	a		10.3
mTOR	a		18.4	a		12.6
Mapk p42/44 cytoplasmic	a		8.6	a		12.0
Cytoplasmic AKT2	a		10.5	c		
Phospho HER2 nuclear	a		10.0	c		
HER2	c			1.43 (1.04, 2.00)	0.03	57.0
Nuclear kRAS	c			a		47.9
Tescy	c			a		44.0
H4jrme	c			a		6.2
Nuclear Mapk	c			a		12.0
Bcl2	c			a		20.7
Tace	c			a		31.8
Tacep	c			a		16.5

Abbreviations: CI=confidence interval; HR=hazard ratio; IQR=inter quartile range; RFS=recurrence-free survival; RfoT=recurrence free while on tamoxifen treatment.

Key: a: Excluded as ‘unreliable’ – inclusion frequency was < 50% samples; b: excluded as ‘unstable’ – form not apparent in ⩾50% samples; c: screened out.

a For biomarkers with linear or polynomial effect, reported HR shows the amount of increase in risk of recurrence per 100 unit changes in the independent variable.

b Before applying cubic transformation to cytoplasmic kRAS, variable was divided by 100.
